# Current professional standing of young medical oncologists in Spain: a nationwide survey by the Spanish Society of Medical Oncology + MIR section

**DOI:** 10.1007/s12094-022-02989-3

**Published:** 2022-11-23

**Authors:** Domingo Antonio Sanchez Martinez, Aliica Quilez-Cutillas, Pablo Jimenez-Labaig, Andrea Sesma, Noelia Tarazona, Vilma Pacheco-Barcia, Berta Obispo, David Paez, Teresa Quintanar, Manuel Sanchez-Canovas, Ana Fernandez Montes, Enriqueta Felip, Alvaro Rodriguez-Lescure, Elena Elez

**Affiliations:** 1 Department of Medical Oncology, IMIB, Virgen de La Arrixaca University Hospital, Murcia, Región de Murcia Spain; 2 Department of Medical Oncology, Can Misses Hospital, Área de Salud de Ibiza y Formentera (ASEF), Ibiza, Illes Balears Spain; 3 Department of Medical Oncology, Cruces University Hospital, Barakaldo, Bizkaia Spain; 4 Department of Medical Oncology, Lozano Blesa University Clinical Hospital, Zaragossa, Aragón Spain; 5 Department of Medical Oncology, INCLIVA Biomedical Research Institute, Comunitat Valenciana, Spain; 6 Instituto de Salud Carlos III, CIBERONC, Madrid, Spain; 7 Department of Medical Oncology, Torrejón University Hospital, Madrid, Spain; 8 Department of Medical Oncology, Infanta Leonor University Hospital, Madrid, Spain; 9 Department of Medical Oncology, Santa Creu i Sant Pau University Hospital, Barcelona, Catalunya Spain; 10 Department of Medical Oncology, Elche University General Hospital, Elche, Comunitat Valenciana Spain; 11 Department of Hematology and Medical Oncology, Morales Meseguer University General Hospital, Murcia, Región de Murcia Spain; 12 Department of Medical Oncology, University Hospital Complex of Ourense (CHUO), Ourense, Galicia Spain; 13 Vall d’Hebron Institute of Oncology (VHIO), Medical Oncology Department, Vall d’Hebron University Hospital, Univesitat Autònoma de Barcelona (UAB), 119-129, 08035 Barcelona, Catalunya Spain

**Keywords:** Career path, Young oncologist, Professional standing, Job performance, Oncology professionals

## Abstract

**Background:**

There is a lack of knowledge about the career paths and employment situation of young medical oncologists. The aim of our study was to evaluate the current professional standing of these professionals in Spain.

**Methods:**

The Spanish Society of Medical Oncology + MIR section conducted a national online survey in May 2021 of young medical oncology consultants (< 6 years of expertise) and final year medical oncology residents.

**Results:**

A total of 162 responses were eligible for analysis and included participants from 16 autonomous communities; 64% were women, 80% were consultants, and 20% were residents. More than half of the participants performed routine healthcare activity and only 7% research activity. Almost three quarters (73%) were subspecialized in a main area of interest and almost half of these chose this area because it was the only option available after residency. Half of the respondents (51%) considered working abroad and 81% believed the professional standing in Spain was worse than in other countries. After finishing their residency, only 22 were offered a job at their training hospital. Just 16% of participants had a permanent employment contract and 87% were concerned (score of ≥ 5 on a scale of 1–10) about their job stability. In addition, one quarter of the participants in our study showed an interest in increasing their research activity.

**Conclusions:**

The choice of subspecialty in medical oncology may depend on job opportunities after residency rather than personal interest. The abundance of temporary contracts may have influenced the job stability concerns observed. Future mentoring strategies should engage in building a long-term career path for young medical oncologists.

**Supplementary Information:**

The online version contains supplementary material available at 10.1007/s12094-022-02989-3.

## Introduction

As it is known, cancer is a major public health problem due to its high incidence and prevalence, and it continues to be one of the leading causes of mortality worldwide. The International Agency for Research on Cancer estimated that, in 2018, some 18.1 million cancers were diagnosed globally [1]. According to world population estimates, by 2040, there will be 30.2 million cases per year [2].

In Spain, the official recognition of medical oncology as a specialty occurred in 1978, becoming one of the first countries in Europe to do so. Medical oncologists (MO) are professionals dedicated to the care and treatment of cancer patients from diagnosis to the final stages of the disease, actively collaborating in the emotional, social, and psychological support of patients and their families. Medical oncology is a demanding, dynamic and evolving medical specialty, which calls for continuous learning and dedication [3]. Acquiring the title of MO requires a training period consisting of 2 years of core training and 3 years of specialized training in medical oncology. Throughout the two periods, the students must develop different competencies, either generic or cross-disciplinary, which are common to all health science specialties [4]. However, knowledge about how to develop a career path and the employment status of young MO is scarce in Spain [5]. The career pathway is a workforce development strategy that integrates programs and services intended to support and develop workers’ academic, technical and employability skills. This strategy is lacking in many countries in Europe, including Spain, but is well adopted in particular countries like the United States [6].

Some problems have been detected in the oncology practice. These problems are often associated with the intrinsic practice of the specialty and at other times are due to external factors (e.g., the work environment, the infrastructure of the department where you are working, the learning process, etc.). Cancer care professionals, especially young oncologists (which includes oncology residents [registrars] and oncology specialists [consultants] in their first 5 years of practice), are at special risk of developing burnout syndrome. This is due mainly to direct contact with seriously ill patients and their families, a continuously changing medical landscape, and significant healthcare pressure [7]. Consequently, young MOs often experience a prolonged state of physical and mental exhaustion that can reduce professional efficacy and result in negative effects on the quality of patient care [8]. The European Society for Medical Oncology (ESMO) recently studied burnout syndrome in young oncologists, showing that measures promoting a good work-life balance, access to support services, and adequate vacation time may reduce burnout levels [9]. The aforementioned situation should be taken into account in the development of the training programs and professional careers of these future oncologists.

In 2021, the Spanish Society of Medical Oncology (SEOM) published the results of a survey on workloads, census, and needs for MOs in Spain [10], and found that almost 40% of participants had a temporary contract and 77% worked in healthcare activities. Furthermore, a strong demand for MOs was observed; however, in order to satisfy this need, long-term career paths would need to be established for young MOs.

In this context, the aim of our study was to evaluate the current professional standing of young MOs in Spain and to seek improvement strategies that could enhance the careers of these professionals.

## Methods

A descriptive observational study was carried out by the SEOM + MIR Section (represented by 12 oncologists, including specialists, residency mentors, and resident medical interns [MIR, *Médico Interno Residente*]), a section devoted to analyzing and addressing the specific concerns of young MOs in Spain. Due to the subject matter of the study and data processing, the study did not require ethics committee approval. We conducted an anonymous online survey between 19 April and 20 May 2021 of young medical oncology consultants (<6 years of expertise) and final year medical oncology residents. Using the electronic mailing available in the SEOM database, professionals from Spain (*N* = 343) were invited to participate in this survey. The survey consisted of 12 multiple-choice and open-ended questions. Participants were not required to answer all questions. Demographic characteristics, employment contracts, professional development, long-term career goals, and concerns about job stability were examined. Questions were also asked regarding mentorship. A copy of the complete survey is shown in Annex 1.

A descriptive analysis of study variables was performed. Categorical variables were expressed as percentages and counts. Data analyses were carried out using IBM SPSS Statistics v27 software (Armonk, NY, USA).

## Results

### Participant demographics

**Fig. 1:**
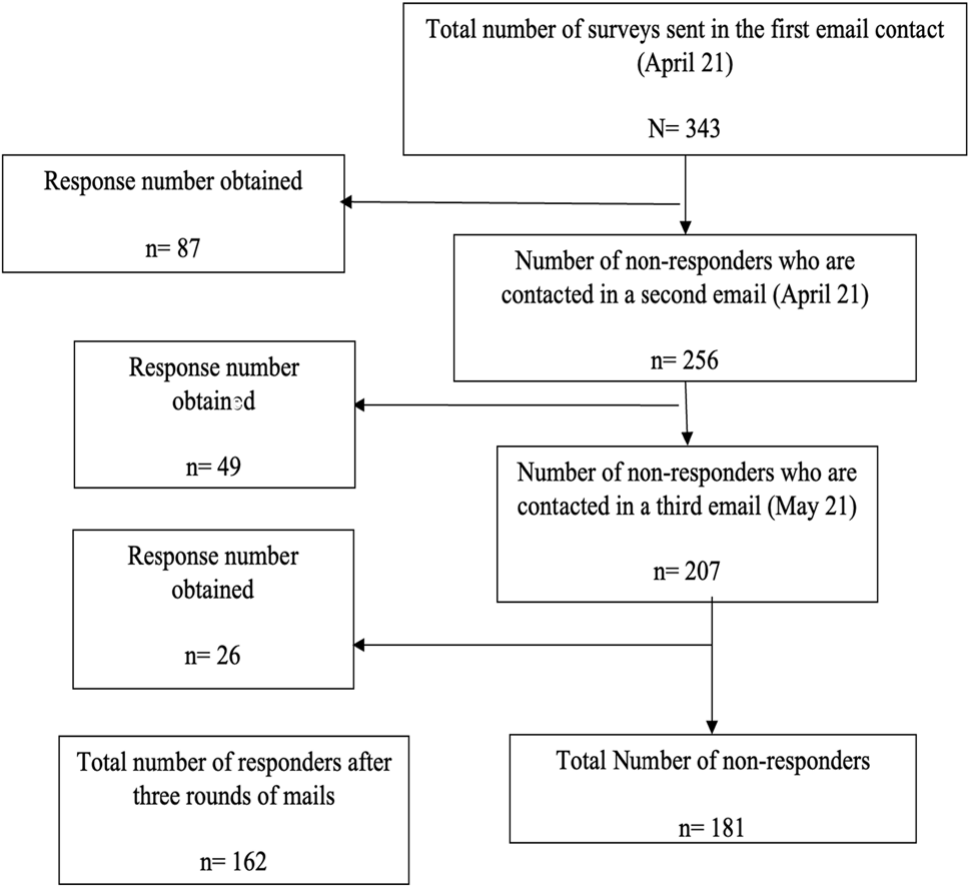
Flowchart of participants’ recruitment

A summary of participant demographics and professional characteristics is shown in Table 1. Of the total number of surveys sent to the target population, we obtained 162 responses in total after three rounds of mails (Fig. 1), 64% of which were from women. The Community of Madrid was the Autonomous Region with most participants (21%), followed by Catalonia and Valencia. In relation to the professional status shown in Table 1, nearly 80% were employed when the questionnaire was conducted. Among these, only 20% had a permanent contract and 23% had signed a COVID19 temporary contract.

**Table 1:** Participant demographics and professional characteristics

	Total *n* (%) *N* = 162 (100.0)
Sex	
Female Male	103 (63.6) 59 (36.4)
Autonomous Community	
Andalusia Aragon Asturias Balearic Islands Canary Islands Cantabria Castile and Leon Castilla La Mancha Catalonia Valencia Estremadura Galicia Madrid Murcia Navarre Basque Country La Rioja Ceuta Melilla	19 (11.7) 5 (3.1) 2 (1.2) 5 (3.1) 5 (3.1) 3 (1.9) 5 (3.1) 3 (1.9) 32 (19.8) 14 (8.6) 3 (1.9) 13 (8.0) 34 (20.1) 7 (4.3) 4 (2.5) 8 (4.9) 0 (0.0) 0 (0.0) 0 (0.0)
Work distribution between participants	
Final year resident (registrar) First Year consultant Second Year consultant Third Year consultant Fourth Year consultant Fifth Year consultant	33 (20.4) 31 (19.1) 28 (17.3) 27 (16.7) 20 (12.4) 23 (12.4)
Position	
Inpatient unit Palliative care Combination of inpatient ward and general oncology clinic Activity set by oncology residency training program Other Unanswered	5 (3.1) 0 (0.0) 14 (8.6) 22 (13.6) 3 (1.9) 118 (72.8)
Professional situation	
Finishing medical residency Employed Permanent contract Temporary contract Unemployed	34 (20.1) 126 (77.8) 26 (16.1) 100 (61.7) 2 (1.2)

### Professional environment and satisfaction

More than two-thirds of participants worked in routine clinical care while only 11 (7%) were involved in research activity. Three-quarters (73%) were subspecialized in a main area of interest, and in more than half of them (59%), this was chosen because it was the only option available after residency. Options such as working in management or in the pharmaceutical industry were not selected by respondents.

Among the total number of doctors surveyed, 54% had considered different employment opportunities other than routine clinical care and almost a quarter showed interest in increasing their research activity. In addition, 32 participants in the sample considered working in the pharmaceutical industry as an alternative.

In relation to the question about working in countries other than Spain, 51% (80 participants) had considered working abroad (56% in countries within the European Union [EU] and 44% outside the EU). Among the different reasons given, 41 thought it might increase their professional development and 33 stated better salary conditions. Furthermore, 132 participants believed the professional standing in Spain was worse than in other countries.

Regarding the person that helped the surveyed oncologists to achieve their professional goals, most said the chief resident, followed by their mentor and tutor. More than a quarter of the respondents stated that they did not obtain any help from the aforementioned figures.

**Fig. 2:**
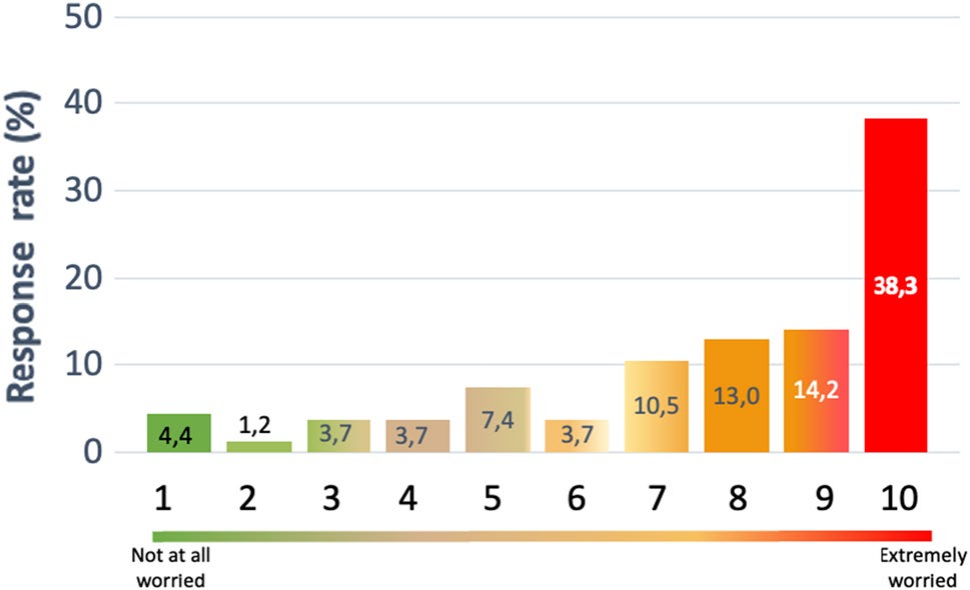
Perceived concerns about job stability among young oncologists

**Fig. 3:**
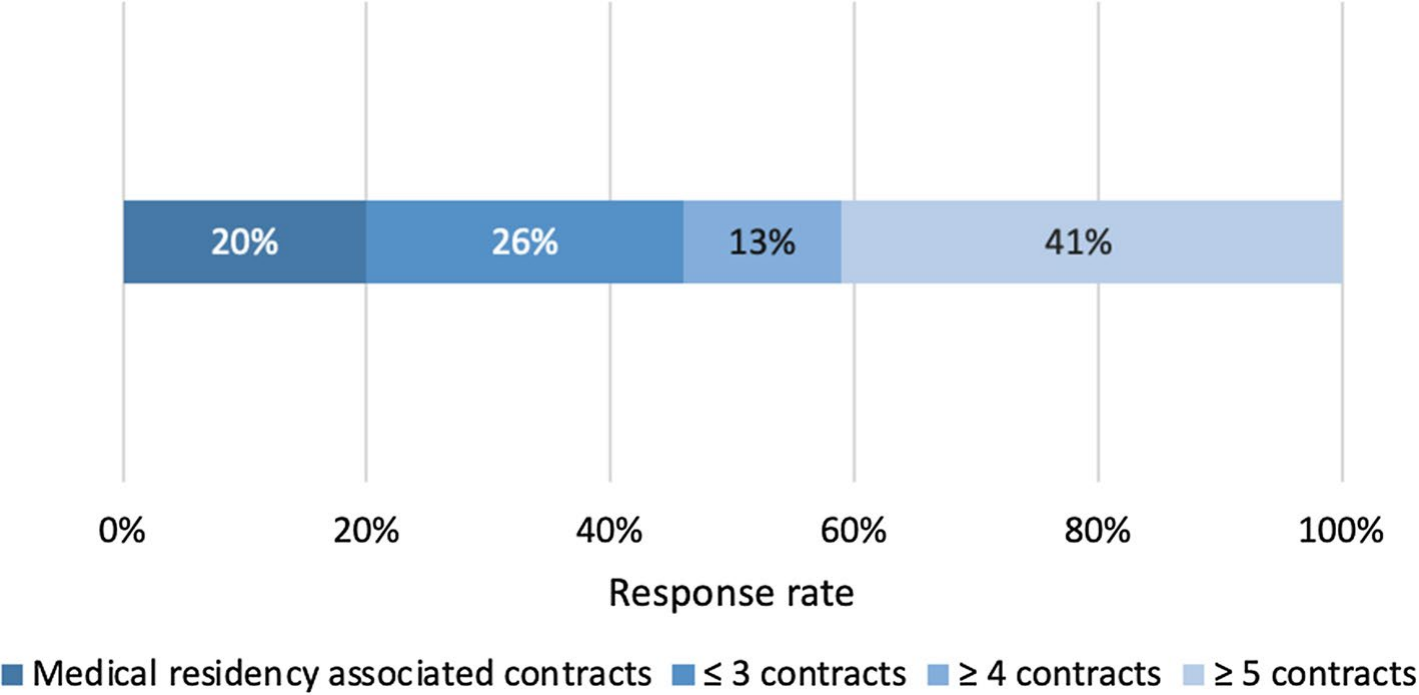
Percentage of respondents according to the number of employment contracts signed in the last 2 years

In order to determine the degree of the precariousness of our respondents, a set of questions was focused on determining their level of professional satisfaction. Forty percent of the population was concerned about their work situation. Figure 2 presents a summary of the findings: 87% were worried about their job stability (scoring ≥ 5 on a scale of 1–10, where 1 = not worried at all and 10 = extremely worried), and almost four in ten were extremely worried. As can be seen in Fig. 3, 41% of the surveyed participants had signed five or more employment contracts in the last 2 years.

### Strategies to improve professional development

When asked about the possibility of participating in a mentoring program focused on helping their professional development, most of the participants (80%) found it a positive strategy, and almost 70% said they would participate.

## Discussion

Our study analyzed the employment situation of young MOs in Spain, finding that the first years after residency present great uncertainty and only a small possibility of subspecializing in the desired area. Up to 59% of newly trained oncologists report having chosen their subspecialty based on job availability, rather than on previous experience, curriculum vitae, or personal interest. The authors consider this a huge obstacle to career development. Another point to note in this respect is that Spain does not have clearly defined subspecialty programs, unlike other countries [11]. As far as the authors are aware, this is the first study published on the aspirations, career prospects, and recruitment data of MOs after completing their training period.

Within clinical care, the complexity of new molecular diagnostic tests and targeted treatments together with complex multidisciplinary management require physicians to have ever more expertise in specific fields of oncology [10]. Subspecialization, with its pros and cons, is the current trend in modern oncology with the creation of different working units [12]. As our population showed, the majority of MOs entered the subspecialty through different routes. However, in this path to modernization, our study demonstrates the importance of chance in becoming subspecialized, taking any vacant contracts offered, as we mentioned.

Subspecialization and the long-term career path are accompanied by extensive and multiple professional profiles that include scientific and clinical knowledge, etc., or variations of these categories, and that have a specific learning curve. While many consider oncology to be a highly specialized and narrow field, it does in fact encompass a wide range of careers that includes (but is not limited to) clinical oncology, research in specific cancers in universities or hospitals, teaching medical students, and any number of combinations and variations thereof [13].

Related to the distribution of the type of work in our population, up to a quarter of the participants were considering increasing their research activity, and half of those looking for new career opportunities would like to include research activity in their working day. However, only 7% were engaged in research in their job, which according to the aforementioned study by the SEOM takes place outside working hours 50% of the time [9]. This trend seems to be contrary to the American trend, where around 60% of research activity has been described among those MOs in the process of subspecializing and is far from the current needs and interest in this area. Furthermore, the absence of participants who reported working in the pharmaceutical industry is striking, an aspect that is addressed in more depth among the limitations of this study, but which, nevertheless, coincides with what has been reported in American studies [14].

A nationwide cross-sectional study carried out in France investigated the main factors affecting the career path choice of oncology residents [6]. These factors were the cross-sectional nature, interest in oncology, variety of human relations and the multi-disciplinary field of work. The preferred career choice was working in a public hospital followed by a career as a doctor/professor involving teaching or research. However, most residents stated that, during their training period, they had not received enough information about future work options and the different potential career paths. The development of a mentoring program, promoting interest in research, and career development during the residency period appears to have a positive influence on career decisions [10]. In addition, this group of medical residents was particularly concerned about their future employment, mainly due to a shortage of openings, heavy workload, and lack of work-life balance [7].

As we can see, working and living conditions are key to setting forth a career path. The present study highlights a clear precariousness in the job stability and financial remuneration of young oncologists compared to the situation of the same professionals in other countries [15]. Almost nine out of ten young oncologists had temporary contracts, 40% of them signing five or more contracts in their first 5 years as specialists. Half of the participants in this study expressed an interest in working abroad (majority in the UE), either to improve their financial situation or to develop professionally. In our view, this poses a serious risk of brain drain, as previously described in other studies [16], as well as higher rates of burnout and job dissatisfaction [17, 18].

In terms of the demographic profile obtained, this showed a significant predominance of female professionals (64%). This represents a slight increase with respect to the total medical oncology specialty in Spain, which stands at 62% in the most recent literature [19]. This figure contrasts with the European average (47.2%) [20] and the US average (35.2%) [21].

Talent flight and work dissatisfaction is compounded by the limited remuneration of healthcare professionals, which is much lower in Spain than in other countries. The average gross earnings in Spain for a physician are $57,000 between the public and private sector, compared to other European countries such as Italy, with salaries of $70,000, France $98,000, England $138,000, and Germany $183,000; farthest from this situation is the USA, with gross earnings of $316,000 [19]. In the case of the youngest oncologist, the income range is $23.000–26.000 [22]. The emigration of doctors and the process of subspecialization are considered to be worse in Europe than further east, according to one study that noted the lack of professionals and high workload [20].

Along the career path, the support and influence of third-party professional partners are invaluable and can be a determining factor [23, 24]. According to those surveyed, 27% indicated that the figure of the chief resident had contributed most to this objective. The mentor, named by 20% of respondents, and the tutor, with 18%, also stand out as figures that have aided the professional development of the oncologists surveyed. Also notable is that up to 27% of the sample reported not having obtained any help from the figures mentioned.

This study also has some limitations. First, it is a retrospective study conducted through a voluntary online survey. This design carries an implicit risk of selection and recall bias in the participants owing to the pull effect to the most affected population by the hiring issues. This study has not evaluated the psychological effect that the lack of work may have on young physicians, in terms of depression and/or anxiety.

However, the results obtained at both the demographic and occupational levels coincide consistently with those described in previous literature, so we do not consider these biases to have a relevant impact. On the other hand, the absence of participants in the present study who reported activities related to the pharmaceutical industry is striking; the authors postulate that this is probably again due to selection bias, as many of these professionals do not belong to the medical society through which the survey was disseminated.

The main strength of this study is that, to the authors’ knowledge, it is the most comprehensive overview to date of the employment landscape of newly specialized MOs. A high participation rate was obtained as well as good representativeness of the different geographical regions of the country among the study participants. Furthermore, the period of data collection coincided with the SARS-COV-2 pandemic emergency situation, which had a significant impact on training and employment in this population [5]. This may undoubtedly influence the study findings, especially regarding aspects such as job stability and the mental health of the professionals. However, there is no doubt that this situation will condition the employment outlook for professionals in the coming years, which is why it is interesting and important to have been able to reflect this situation in the study. Some studies have begun to point the way towards the competency profiles of these future professionals [20].

## Conclusion

This study is the first to analyze the aspirations and career prospects of oncologists after completing their residency, showing that special involvement and interest from stakeholders is needed to improve oncology now and in the future. An absence of engagement in the long-term career paths of young MOs in Spain was observed. Furthermore, there is a lack of contractual quality and planning for entry into the labor market of newly trained oncologists. Further studies are needed to substantiate concrete measures at the national level to improve the working conditions of the future providers of medical oncology services.

## Supplementary Information

Below is the link to the electronic supplementary material.

Supplementary file1 (DOCX 14 kb)
