# Supplementary material for: Current professional standing of young medical oncologists in Spain: a nationwide survey by the Spanish Society of Medical Oncology + MIR section
Source: Clin Transl Oncol. 2022 Nov 23;25(3):796–802. doi: 10.1007/s12094-022-02989-3 (PMC9685015; doi:10.1007/s12094-022-02989-3)
Supplement: Supplementary file 1 — Supplementary file1 (DOCX 14 kb) [file 12094_2022_2989_MOESM1_ESM.docx]

**Annex I.- Work Thermometer Survey of Oncology in Spain: present and future of young oncologists.**

**1.1. Affiliation:**

**1.1-Year of residence/attachment** (R5-A5): R5-A1-A2-A3-A3-A4-A5 (choose one of the 6 options).

**1.2-Province of professional development:** (list provinces)

**2.-Current employment situation:**

**2.2.1 What is your main professional activity?**

-Clinical care

-Research

-Pharmaceutical industry

-Management

-Work not related to oncology (add box to be filled in)

**2.2.2 What type of contract do you have?**

-Permanent

-Temporary

**2.2.3 What is the weekly duration of your contract?**

-40 hours per week

-37.5 hours per week

-Less than 37.5 hours per week.

**2.2.4 Of these contract hours, what is the main purpose of the contract?**

-Clinical care

-Research

-Mixed

**2.2.5 Do you consider that this distribution is accomplished?**

-Yes

-No

**2.2.4 Have you signed a Covid contract?**

-Yes

-No

*** 2.1 Have you been offered to stay in your department?**

-Yes

-No

***2.2 How did you get your job?**

-Job vacancies

-Presentation of CV to the head of department

-Scholarships

**3.-How much do you worry about your job stability? (Job stability understood as the possibility of remaining in the same job for more than one year)** ** (Job stability understood as the possibility of remaining in the same job for more than one year)**

-1 to 10

**4.-How many employment contracts have you signed in the last two years?**

-1

-2

-3

-4

-5 or more

**5.-Have you considered other employment opportunities apart from clinical care?**

-No

-Yes, working in the pharmaceutical industry

-Yes, working exclusively in research

-Other

**6.1 Have you considered working abroad?**

-No

-Yes*

**6.1.1 If you answered yes, where?**

-*Within the European Union

-Outside the European Union

**6.1.2 Why?**

-Better pay conditions

-Professional development

-Personal reasons

-Other (free text)

**7.-How do you see the employment situation in oncology in Spain compared to other countries?**

-Better

-Worse

-The same

-Don’t know

**8.-If you are a young registrar, do you consider yourself in a precarious situation?**?**

-Yes

-No

-I am not a registrar yet.

**9.-Do you see your job expectations fulfilled after finishing your residency?**

-Yes

-No

**9.1 **Add this question if you tick NO. Question: Why (select one or more)?**

-Non-competitive salary

-Lack of job progression.

-Time for patient care/research/teaching is not respected.

-Pandemic situation due to Covid.

-Other (free text)

**10.-Do you know if any of the following processes have been carried out in your Autonomous Community in the last 5 years? Indicate the ones you know of that have been carried out.**

-Public Employment Offer for your specialty.

-Process of consolidation of job positions.

-No actions aimed at access to stable job positions have been carried out.

-I don't know if the abovementioned actions have been carried out.

**11. Who do you consider to have helped/supervised you so that you could develop as an oncologist and enhance your professional career?**

-Chief registrar/consultant

-Mentor (person who has advised or guided you in a certain career path) -Sponsor (person who has helped you to develop your career)

-Sponsor (person who has offered you a job opportunity) -Sponsor (person who has offered you a job opportunity)

**12. Would you be interested in participating in this mentoring programme?**

-Yes

-No
